# Supplementary figures and images for: Identification of Novel Biomarkers in Late Preterm Neonates with Respiratory Distress Syndrome (RDS) Using Urinary Metabolomic Analysis
Source: Metabolites. 2023 May 9;13(5):644. doi: 10.3390/metabo13050644 (PMC10223367; doi:10.3390/metabo13050644)

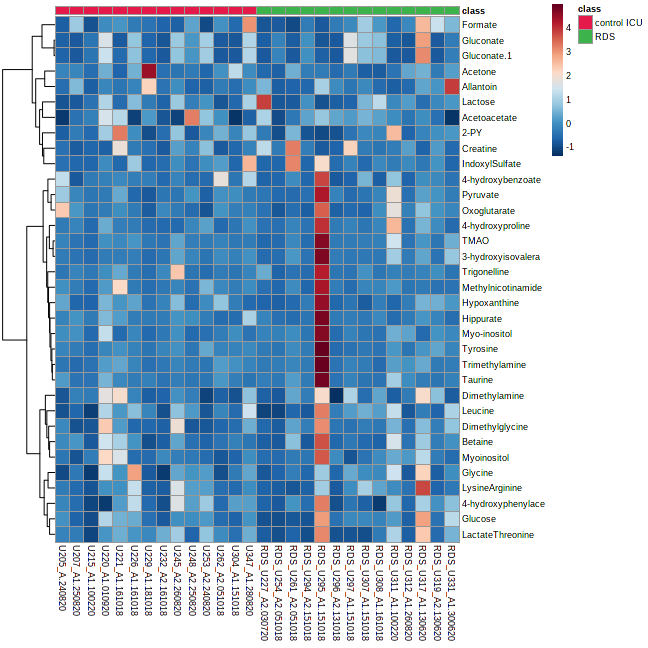

Supplement: Supplementary file 1 [file metabolites-13-00644-s001.zip › Figure S1.tiff]

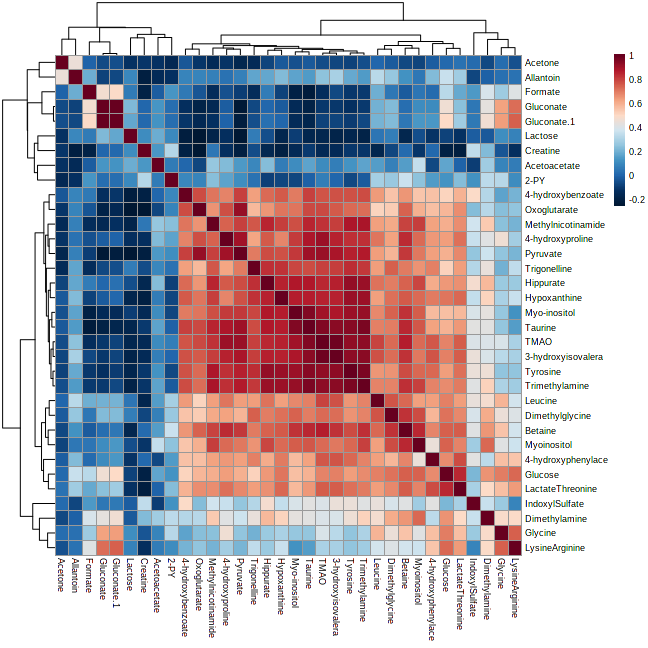

Supplement: Supplementary file 1 [file metabolites-13-00644-s001.zip › Figure S2.tiff]

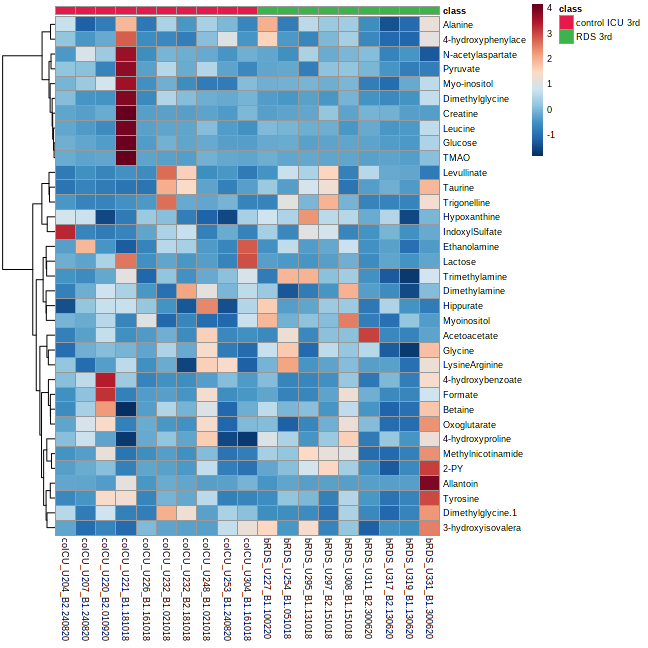

Supplement: Supplementary file 1 [file metabolites-13-00644-s001.zip › Figure S3.tiff]

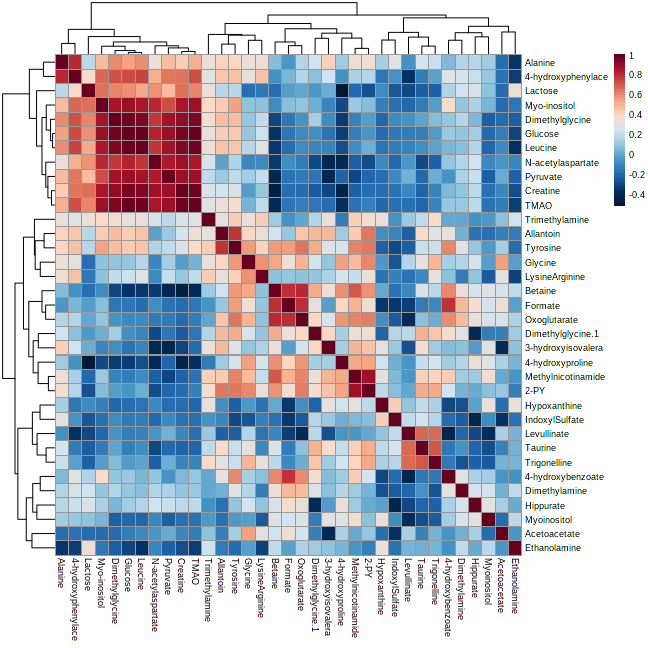

Supplement: Supplementary file 1 [file metabolites-13-00644-s001.zip › Figure S4.tiff]

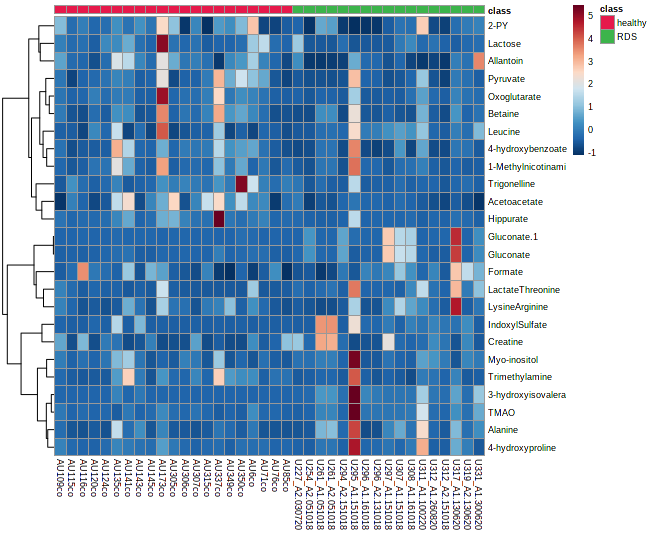

Supplement: Supplementary file 1 [file metabolites-13-00644-s001.zip › Figure S5.tiff]

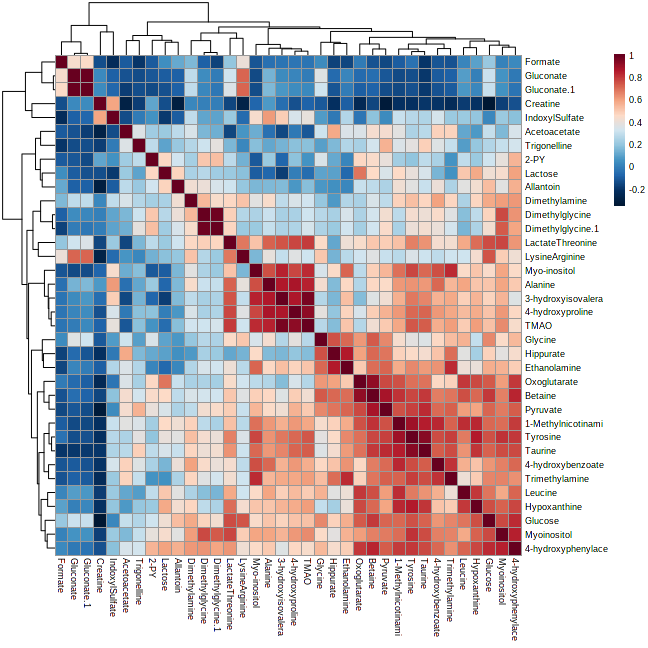

Supplement: Supplementary file 1 [file metabolites-13-00644-s001.zip › Figure S6.tiff]

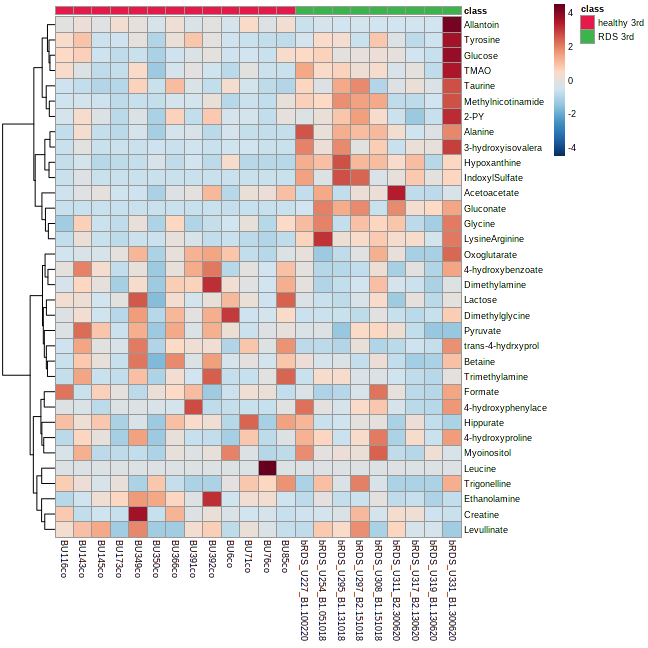

Supplement: Supplementary file 1 [file metabolites-13-00644-s001.zip › Figure S7.tiff]

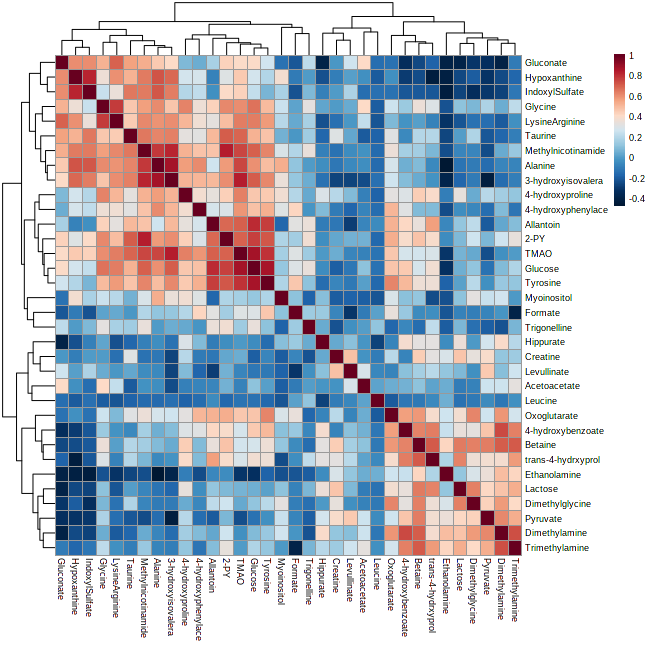

Supplement: Supplementary file 1 [file metabolites-13-00644-s001.zip › Figure S8.tiff]
